# Grassland dynamics in response to climate change and human activities in Xinjiang from 2000 to 2014

**DOI:** 10.1038/s41598-018-21089-3

**Published:** 2018-02-13

**Authors:** Renping Zhang, Tiangang Liang, Jing Guo, Hongjie Xie, Qisheng Feng, Yusupujiang Aimaiti

**Affiliations:** 1 Institute of Arid Ecology and Environment, Key Laboratory of Oasis Ecology, Xinjiang University, Urumqi, 830046 China; 2 State Key Laboratory of Grassland Agro-ecosystems, College of Pastoral Agriculture Science and Technology, Lanzhou University, Lanzhou, 730020 China; 3 Xinjiang Academy Forestry, Urumqi, 830000 China; 4 Department of Geological Sciences, University of Texas at San Antonio, Texas, 78249 USA; 5 Department of Urban Environment Systems, Chiba University, Chiba, 263-8522 Japan

## Abstract

Climate change and human activities are two key factors that affect grassland ecosystem. Accurately estimating the effects of these two factors on grassland dynamics and understanding the driving forces of the dynamics are important in controlling grassland degradation. In this study, the potential Net Primary productivity (NPP_P_) and the difference between NPP_P_ and actual NPP (NPP_A_) are used as indicators of climate change and human activities on grassland ecosystem in Xinjiang. An overall grassland NPP_A_ increase than decrease (69.7% vs 30.3%) is found over the study period of 2000 to 2014. While human activities played a dominant role for such a NPP_A_ increase, both human activities and climate change contributed almost equally to the grassland NPP_A_ decrease. Within the three types of grasslands in Xinjiang, the desert grassland showed the greatest NPP_A_ increasing trend that mostly attributed to human activities; the meadow showed an overall NPP_A_ decreasing trend that was mainly caused by human activities; the steppe showed similar NPP_A_ decreasing and increasing trend in terms of area percentage. Based on this study, our recommendations are (1) to continue the grazing prohibition policy in desert grassland and (2) to extensively implement the rest grazing policy in steppe and meadow grasslands.

## Introduction

The International Biology Program (IBP), Global Change and Terrestrial Ecosystems (GCTE), International Geosphere-Biosphere Program (IGBP) and the Kyoto Protocol made the terrestrial environment change as one of their core research projects^1^. The dynamic changes in terrestrial ecosystems are the consequences of both climate change and human activities. With the warming climate and intensified human activities over the past decades, surface vegetation has deviated from its steady state and formed a typical spatiotemporal patterns and change processes^2,3^. Despite the fact that terrestrial ecosystem changes could be accurately monitored, there are disagreements about the driving factors and their individual importance to the terrestrial ecosystem changes^1,4–6^. Especially in arid and semi-arid regions, climate change and intensified human activities could easily lead to ecological degradation and even cause serious ecological and economic losses^7^. In general, grassland degradation includes the degradation in its quality, productivity, economic potential, biological diversity or complexity^8^. Therefore, accurately measuring the impacts of climate change and human activities on the ecosystem changes has a significant importance in developing effective policies for ecological management and regulation at both national and global scales^9,10^.

Grassland ecosystem, as one of the important terrestrial ecosystems, plays an important role in regulating global carbon cycle and climate. Meanwhile, grassland ecosystem is also related to the ecological and environmental conditions of its surrounding areas, as well as the socio-economic development and regional ecological safety. In recent years, with the increase in temperature and precipitation, plus implementation of various programs for ecological protection at local, regional and national levels, increase of grassland net primary productivity in some areas of China has been reported^8,11,12^. In contrast, decrease of grassland net primary productivity, due to the dry climate and overgrazing, was also reported^13,14^; the impacts of climate change on grassland vegetation were also found with differences in terms of grassland types and seasons^15^. Moreover, due to the increasing human disturbances, the trend of future grassland ecosystem change may become even more complex.

The grassland in Xinjiang is ranked as the third largest grassland in China, accounting for 34.43% of the total land area in Xinjiang^16^. As an arid and semi-arid region, the ecosystem in Xinjiang is extremely fragile, and the grassland ecosystem is generally more fragile and/or sensitive to climate change and human activity (i.e., overgrazing, amelioration, grazing prohibition, rest grazing and grassland reclamation)^17,18^. Moreover, the impacts of climate change and human activity showed a great spatial heterogeneity among different vegetation types in Northern Xinjiang^19^. Hence, the local government has been facing difficulties in determining how to manage the grassland in such a complex environment and how to determine the degree to which grassland has been affected by human activities and/or climate change. Thus, an effective method is needed for addressing the impacts of climate change and human activities on different grassland types.

At present, two types of methods have been used to calculate the relative effects of human activities on grassland ecological change. The first method is to use regression analysis or principal component analysis in determining the relative importance of meteorological, social, and economic factors on grassland primary productivity^20,21^. However, this method emphasizes more on the regression coefficients and rates of variance that represent the relative importance on climate change and human activity, while ignores their real ecological significances in a qualitative way^21^, easily leading to large uncertainties of the results.

The second method is to use remotely sensed indices of higher spatial and temporal resolutions in determining the importance of each factor of climate and human activities on the grassland dynamics; commonly the net primary productivity (NPP) and normalized difference vegetation index (NDVI) are used. The NPP is the amount of solar energy converted to chemical energy by vegetation through the process of photosynthesis and represents the total amount of organic matter accumulated by vegetation per unit area and time; it is an important parameter of ecosystem functioning and the carbon cycle^22^. Many researchers have attributed the NPP as an indicator to reflect the role of climate change and human activities on terrestrial vegetation^23–25^ and one of the key elements of carbon cycle^12^. The potential NPP (NPP_P_) and the difference between the NPP_P_ and actual NPP (NPP_A_) were used as indicators for the impact of climate change and human activities on grassland dynamics^26,27^.

In this study, the above-mentioned second method is used to study the Xinjiang grassland changes and their causes, with three objectives: (1) analyze the spatiotemporal dynamics of grassland NPP_P_ and NPP_A_; (2) evaluate the relative effects of climate change and human activities on the changes of grassland dynamics; and (3) provide potential strategies for future grassland restoration and management.

## Results

### Trends of NPP_A_ change

**Figure 1:**
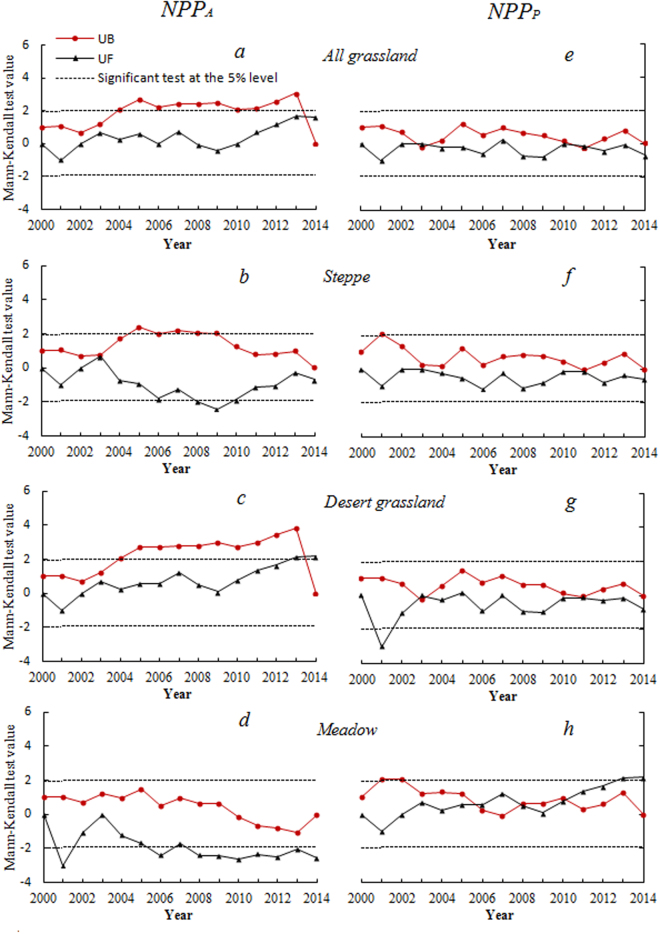
Spatial change trends of Xinjiang grassland NPP_A_ (**a**) and (NPP_P_) (**b**) from 2000–2014. The maps were generated by ArcGIS 10.2, URL:286 http://support.esri.com/Products/Desktop/arcgis-desktop/arcmap/10-2-2#overview.

In the study area, the model-simulated grassland NPP_A_ shows an increasing trend over majority (69.7%) of the grassland in Xinjiang (Fig. 1a). Among them, 16.2% shows significant increase, mainly distributed along the edge of the Tarim Basin and southern margin of the Junggar Basin; 53.5% shows increase but not significant, mainly distributed in the Junggar and Tarim basins. About one third (30.3%) of grassland shows decreasing trend, including 10.7% as significant decrease, mainly distributed in the Ili river valley and the Altai mountains.

### Trends of NPP_P_ change

Climate-induced potential NPP_P_ shows a slightly smaller percentage of grassland increase (64.8%) as compared with the NPP_A_ (69.7%) (Fig. 1b), with only 3% grassland with significant increase, mainly distributed on Kunlun Mountain. Other grassland with increased NPP_P_ (61.8%) but insignificant are mainly distributed in Junggar Basin, Tarim Basin and Altai Mountain. Of the 35.2% grassland with decreased NPP_P_ , only 2.1% shows significant decrease mainly distributed in the Ili river valley. The other 33.1% shows decrease but insignificant, mainly distributed on the margin of Junggar Basin and Tarim Basin adjacent to Tianshan Mountain. However, these results are only an ideal situation caused by climate change without any human interaction.

### M-K abrupt points analysis

**Figure 2:**
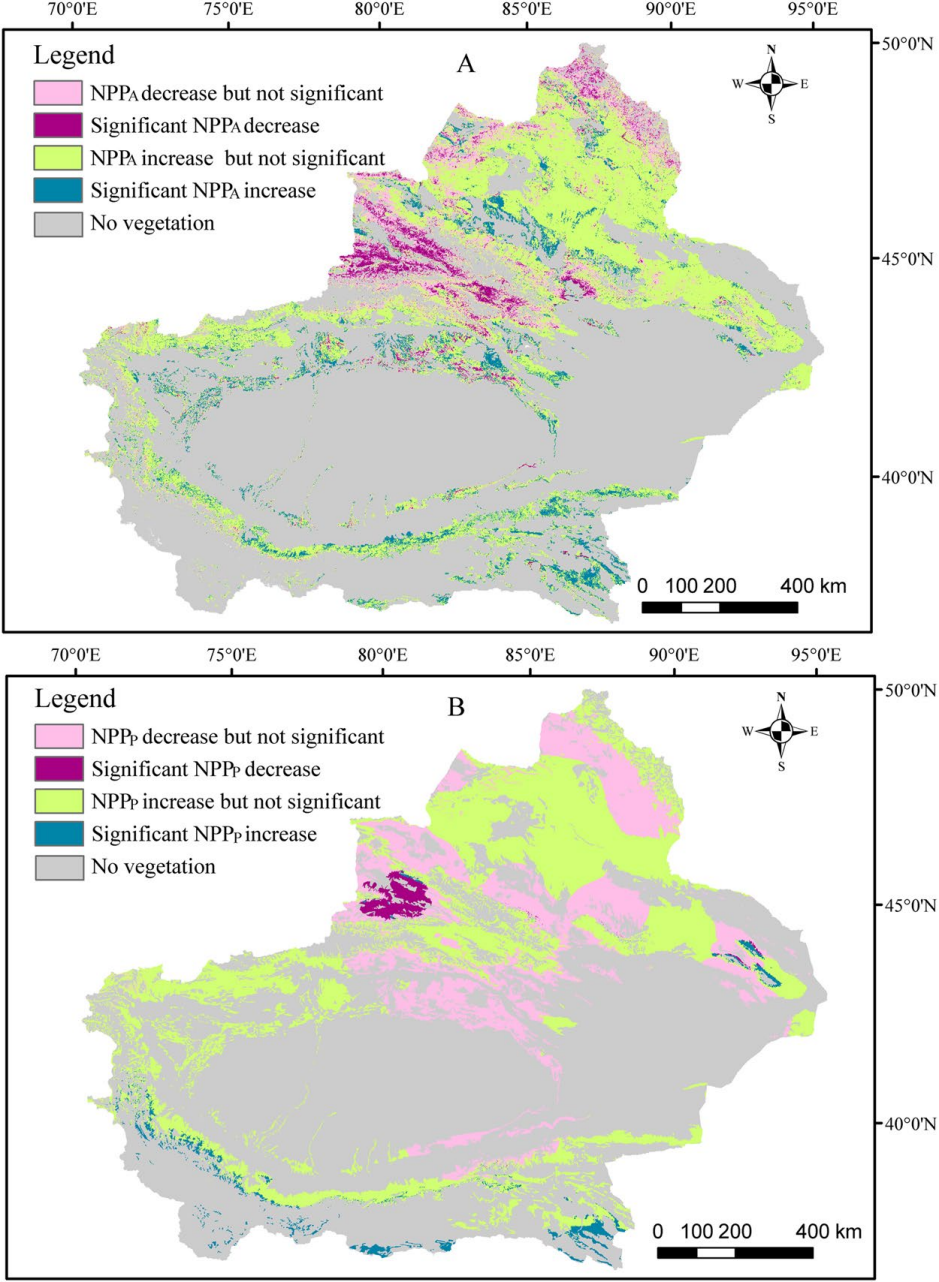
Mann-Kendall mutation criterion curve of average NPP_A_ (left panel) and NPP_P_ (right panel) in Xinjiang grassland from 2000–2014. (**A** and **E**) Mean Mann-Kendall mutation criterion of average NPP_A_ and NPP_P_ of overall grassland; (**B** and **F**) mean Mann-Kendall mutation criterion of average NPP_A_ and NPP_P_ of steppe grassland; (**C** and **G**) mean Mann-Kendall mutation criterion of average NPP_A_ and NPP_P_ of desert grassland; (**D** and **H**) mean Mann-Kendall mutation criterion of average NPP_A_ and NPP_P_ of meadow grassland.

The MK test shows some abrupt points on the mean NPP_A_ and NPP_P_ trends from 2000–2014 (Fig. 2). From the actual NPP (NPP_A_), an overall grassland improvement is indicated (Fig. 2A), although it is insignificant at 95% level. However, after 2013, there is a mutation point, which suggests that the grassland NPP_A_ increase became more obvious. For the steppe grassland (Fig. 2B), a significant NPP_A_ decrease before increase in 2009 is found. For the desert grassland (Fig. 2C), a NPP_A_ increase is clearly seen since 2000 and such increase became significant in 2013. This is also suggested by the mutation point seen after 2013. For the meadow grassland (Fig. 2D), an overall significant NPP_A_ decrease is clearly seen.

For the potential NPP (NPP_P_), however, there is no apparent abrupt points, except for the meadow grassland (Fig. 2H), a clear significant increase found in 2013.

### The reasons of NPP_A_ change

**Figure 3:**
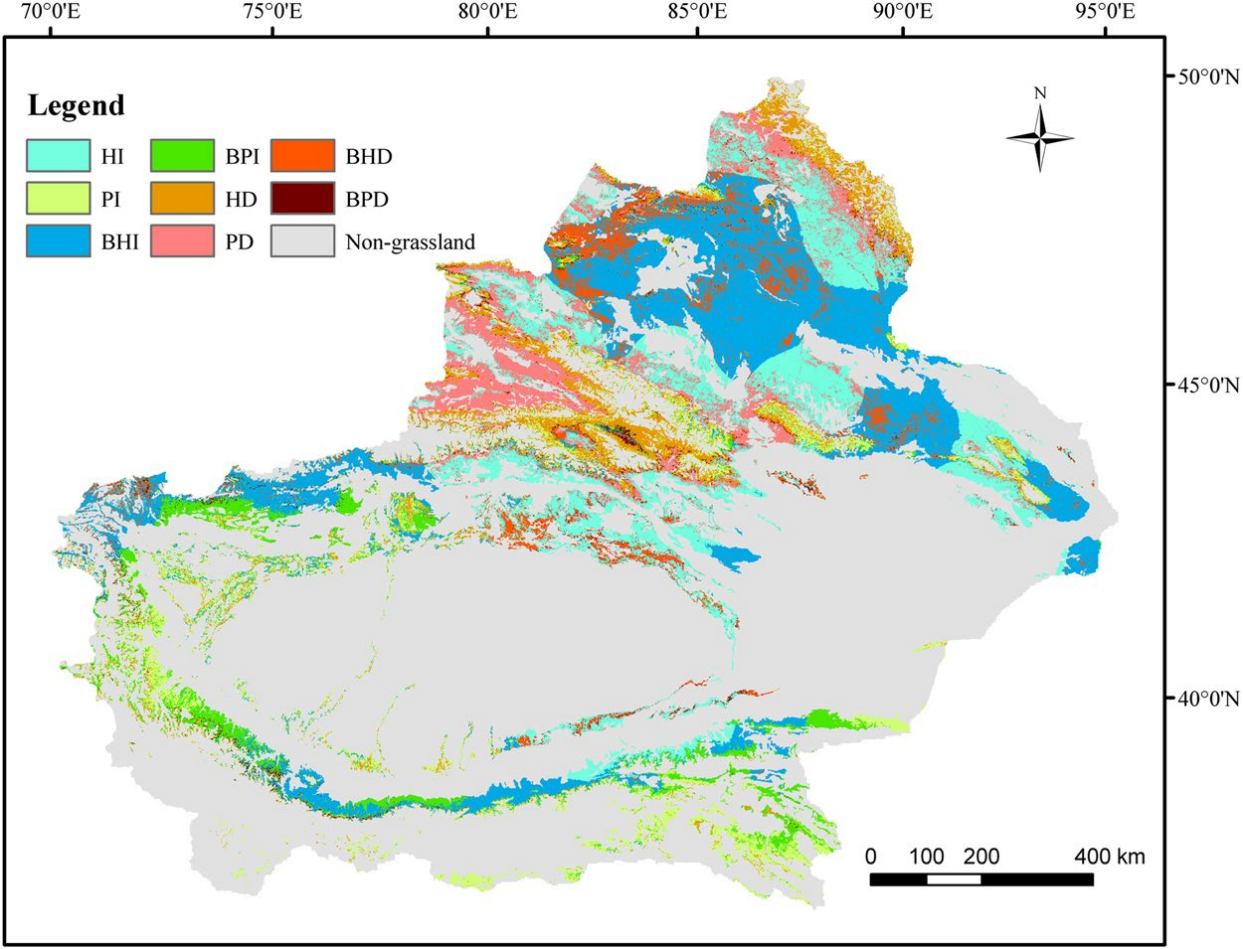
Relative effects of climate change and human activities on grassland changes in Xinjiang from 2000–2014. HI denotes human activities induced grassland NPP_A_ increase; BHI denotes both two factors induced, but human activity dominated grassland NPP_A_ increase; PI denotes climate change induced grassland NPP_A_ increase; BPI denotes both two factors induced, but climate change dominated grassland NPP_A_ increase; TI denotes total grassland NPP_A_ increase; HD denotes grassland NPP_A_ decrease induced by human activities; BHD denotes both two factors induced, but human activities dominated grassland NPP_A_ decrease; PD denotes grassland NPP_A_ decrease induced by climate change; BPD denotes both two factors induced, but climate change dominated grassland NPP_A_ decrease; TD denotes total grassland NPP_A_ decrease. The maps were generated by ArcGIS 10.2, URL:286 http://support.esri.com/Products/Desktop/arcgis-desktop/arcmap/10-2-2#overview.

Climate change and human activities have led to the pattern changes of the Xinjiang grasslands, but their relative effects are different in time and space. Table 1 shows the relative effects of climate change (SlopeNPP_P_) and human activities (SlopeNPP_H_) on grassland change (SlopeNPP_A_) in six different scenarios. Based on the 6 possible scenarios of climate change and human activities (Table 1), the relative roles of them are shown in Fig. 3 and Table 2. The reasons for grassland NPP_A_ decrease or increase have apparent dissimilarities. For example, the human-induced total grassland NPP_A_ increased area accounts for 53.1% of the total 69.7% NPP_A_ increased area and are mainly distributed on the margins of the Junggar Basin, Altai Mountain, adjacent areas to the Junggar Basin, some local areas in the Kunlun Mountains, and eastern Xinjiang region. Climate-induced total grassland NPP_A_ increase only accounts for 16.6% of the total 69.7% NPP_A_ increased areas and are mainly in the surrounding areas of Tarim Basin, except the northeast of the Basin. Only 30.3% of grassland shows NPP_A_ decrease, with human activity-induced NPP_A_ decrease accounted for 16.7%, slightly higher than the 13.6% induced by the climate change. These NPP_A_ decreased areas are mostly distributed in the northern Xinjiang, including the central Altai mountains, the Ili river Valley (mostly climate-induced), the Junggar Basin (mostly human-induced), and the southern slope of Tianshan Mountain (mostly human-induced).

**Table 1:** Evaluation methods of climate change and human activities on grassland dynamics under six possible scenarios.

	*SlopeNPP*_*A*_	*SlopeNPP*_*P*_	*SlopeNPP*_*H*_	Relative role of climate change (%)	Relative role of human activity (%)	Description
Scenario1	+	−	−	0	100	Human activity (100%) contributes grassland NPP_A_ increase.
Scenario2	+	+	−	$\frac{|SlopeNP{P}_{P}|}{|SlopeNP{P}_{P}|+|SlopeNP{P}_{H}|}$	$\frac{|SlopeNP{P}_{H}|}{|SlopeNP{P}_{P}|+|SlopeNP{P}_{H}|}$	Both climate change and human activity contribute NPP_A_ increase.
Scenario3	+	+	+	100	0	Climate change (100%) contributes grassland NPP_A_ increase.
Scenario4	−	+	+	0	100	Human activity (100%) contributes grassland NPP_A_ decrease.
Scenario5	−	−	+	$\frac{|SlopeNP{P}_{P}|}{|SlopeNP{P}_{P}|+|SlopeNP{P}_{H}|}$	$\frac{|SlopeNP{P}_{H}|}{|SlopeNP{P}_{P}|+|SlopeNP{P}_{H}|}$	Both climate change and human activity contribute NPP_A_ decrease.
Scenario6	−	−	−	100	0	Climate change (100%) contributes grassland NPP_A_ decrease.

**Table 2:** Changes of grassland induced by climate change and human activity (Units: km^2^, %).

		Steppe	Desert grassland	Meadow	Xinjiang
HI	area	7392	84747	1439	93578
	percent	8.7	25.9	2	19.3
BHI	area	15687	145016	3004	163707
	percent	18.5	44.2	4.2	33.8
HI + BHI	area	23079	229763	4443	257285
	percent	27.2	70.1	6.2	53.1
PI	area	11518	16669	15599	43786
	percent	13.6	5.1	21.8	9
BPI	area	8325	25066	3093	36484
	percent	9.8	7.6	4.3	7.5
PI + BPI	area	19843	41735	18692	80270
	percent	23.4	12.7	26.1	16.6
TI	area	42922	271498	23135	337555
	percent	50.6	82.8	32.3	69.7
HD	area	10234	2830	32819	45883
	percent	12.1	1	45.9	9.5
BHD	area	9832	22404	2553	34789
	percent	11.6	6.8	3.6	7.2
HD + BHD	area	20066	25234	35372	80672
	percent	23.7	7.8	49.5	16.7
PD	area	18759	29190	11342	59291
	percent	22.1	8.9	15.9	12.2
BPD	area	3078	1788	1671	6537
	percent	3.6	0.5	2.3	1.4
PD + BPD	area	21837	30978	13013	65828
	percent	25.7	9.4	18.2	13.6
TD	area	41903	56212	45832	143947
	percent	49.4	17.2	67.7	30.3

Note: HI denotes human activities induced grassland NPP_A_ increase; BHI denotes both two factors induced, but human activity dominated grassland NPP_A_ increase; PI denotes climate change induced grassland NPP_A_ increase; BPI denotes both two factors induced, but climate change dominated grassland NPP_A_ increase; TI denotes total grassland NPP_A_ increase; HD denotes grassland NPP_A_ decrease induced by human activities; BHD denotes both two factors induced, but human activities dominated grassland NPP_A_ decrease; PD denotes grassland NPP_A_ decrease induced by climate change; BPD denotes both two factors induced, but climate change dominated grassland NPP_A_ decrease; TD denotes total grassland NPP_A_ decrease.

In terms of three different types of grassland, their changes and relative reasons of change are different. For example, the NPP_A_ decreased and increased area percentages of the Steppe are very similar (~50% each), with similar impacts from both the climate and human activities (~25% each); however, the Desert grassland NPP_A_ has a totally different picture, with 82.8% area showing increase, and the human activities account for 70.1% of it. In contrast, a total 67.7% of Meadow NPP_A_ shows decrease, with human activities contributed 49.5% of this decrease. Of the 32.3% meadow NPP_A_ increase, climate change contributes 26.1% of it.

## Discussion

### NPP_A_ and NPP_P_ change in Xinjiang

The Miami model-simulated NPP_P_ is only influenced by climatic factors and has been regarded as the maximum NPP of an ecosystem^28^. In an arid and semi-arid region, the vegetation NPP is more sensitive to precipitation and vulnerable to climate warming^27,28^. According to the fifth report of IPCC, in the past three decades, global temperatures and precipitation have shown an increasing trend. Gao *et al*.^28^, compared the average annual values of the normalized difference vegetation index (NDVI) with theoretical NPP values based on Miami Model to determine the effect of historic climate change on global grassland productivity from 1982 to 2011. They found an increase in most of the grassland NPP_P_, and concluded the global grassland areas have been significantly affected by climate change. In this study, the grassland NPP_P_ in Xinjiang also showed an overall increasing trend. The increase may be related to the fact that, in the past ten years, the average annual temperature and precipitation in Xinjiang have increased, showing an overall trend of warm - wet climate^17^. Chen *et al*., analyzed the impact of climate change and anthropogenic activities on alpine grassland over the Qinghai-Tibet Plateau from 1982 to 2011, and found an increasing trend of NPP_A_ for the entire period, while an increasing trend of NPP_P_ during 1982 to 2001 and decreasing trend of NPP_P_ during 2001 to 2012. As the annual precipitation is the main driving force of the alpine grassland NPP_P_ , the NPP_P_ decline since 2000 was due to the rainfall decrease^29^. This further indicates that, the variability in actual and potential productivity may different throughout the global grassland ecosystems, and may show various trends in different grassland regions, since human activities and climate change present various contributions to the productivity of grassland in different regions.

### Impacts of climate change and human activities on grassland changes in Xinjiang

Detection of the change in terrestrial ecology, based on long-term observation and remote sensing data, has gained a lot of progresses in recent decades^30,31^. However, it is still a complex and challenging task to separate the effects of human activities from those of climate factors^1,28^. Chen *et al*., found that during 2001–2011, human activities were the dominant factors for the improvement of grassland productivity, plus the implemented grassland conservation policies that played a very important role in the grassland restoration^29^. This is consistent with Fang *et al*., who found that the human activities caused the grassland degradation in Qinghai Province during 1990–2000. Since 2000, human activities have gradually played a positive role in the restoration of Grasslands^32^. Our results suggest that majority of the grassland in Xinjiang show NPP_A_ increase from 2000–2014, and the human activities were the dominant factor for such increase (53.1% of the total 69.7% NPP_A_ increased area).

Although previous studies^17,33^ had similar conclusion in terms of grassland NPP_A_ increase in Xinjiang, none of them could tell how much human activities played as compared with climate change for the NPP_A_ increase. Specifically, based on the MK test of this study, there was not much difference in terms of contributions of climate and human activities to the grassland change before 2005, while human activities has become the dominant role for the overall grassland NPP_A_ increase since 2005. This finding has significant implication in terms of (1) understanding the regional difference of climate change to ecological environment change, (2) the important role of human activities and management to such change, in particularly, to the arid grassland area, and (3) the importance of accurately assess climate and human impacts on different environments for better policy making.

### Grassland NPP_A_ increase benefited from effective policy

The change in trends for NPP_A_ and NPP_P_ in different grassland areas are quite different (Fig. 1). In the border areas of Junggar Basin, Tianshan Mountain and Altai Mountain, NPP_P_ shows a reducing trend, while the NPP_A_ shows an increasing trend that could have been related to the recent years’ grassland ecological protection projects^29,32,34^.

For example, return grazing land to grassland project was started to implement in 2003, starting with concrete regulations including the prohibition of desert grassland grazing, and re-vegetation with appropriate rest periods for the steppe and meadow grasslands^25^. In the same time, to ensure that livestock have enough food to eat in winters, grasses were planted in areas of favorable thermal conditions and fertile soil, to complement potential forage shortage in winters.

The NPP_P_ of the grassland in the Altai Mountains shows an increasing trend, while the NPP_A_ shows a significant decreasing trend. This could be related to overgrazing in that area^18^. Although regulations were implemented for meadow re-vegetation with appropriate rest periods, project implementation was difficult due to the high altitude. Additionally, the livestock exiled from the desert grassland were moved to the meadow, increasing the pressure from grazing in those regions. This led the NPP_A_ to decrease in the meadow. Meanwhile, our research shows that the grassland in the Altai Mountains and Ili river valley has a decreasing trend, which is consistent with the conclusions of other studies^18,35^.

Based on our finding from this study, below are our recommendations for grassland’s further recovery. (1) For the desert grassland, grazing prohibition should be continued in this region. (2) Although the steppe and meadow partially implemented the rest grazing policy, the changing trend of the two grasslands are significantly different. NPP_A_ decrease trend in meadow is obvious; the NPP decreased area accounts for 67.7% of the meadow area, in which, 49.5% was due to human activities. The steppe NPP_A_ decreased area accounts for 49.4% of the steppe area, in which 23.7% was due to human activities. The reason for the larger NPP_A_ decrease in meadow could be mainly due to its higher altitude, which made the implementation of the grassland project more difficult. For example, the fencing project was not completed, resulted in that the livestock transferred to the high altitude meadow, some to steppe. This increased the grazing pressure to these two types of grasslands, especially of the meadow land. Therefore, in order to reduce the grazing pressure in these two types of grassland, the number of livestock should be appropriately reduced, and grass planting should be developed in areas with better light, water and heat resources, and herder settlement projects should be extensively implemented.

## Methods

### Study area

**Figure 4:**
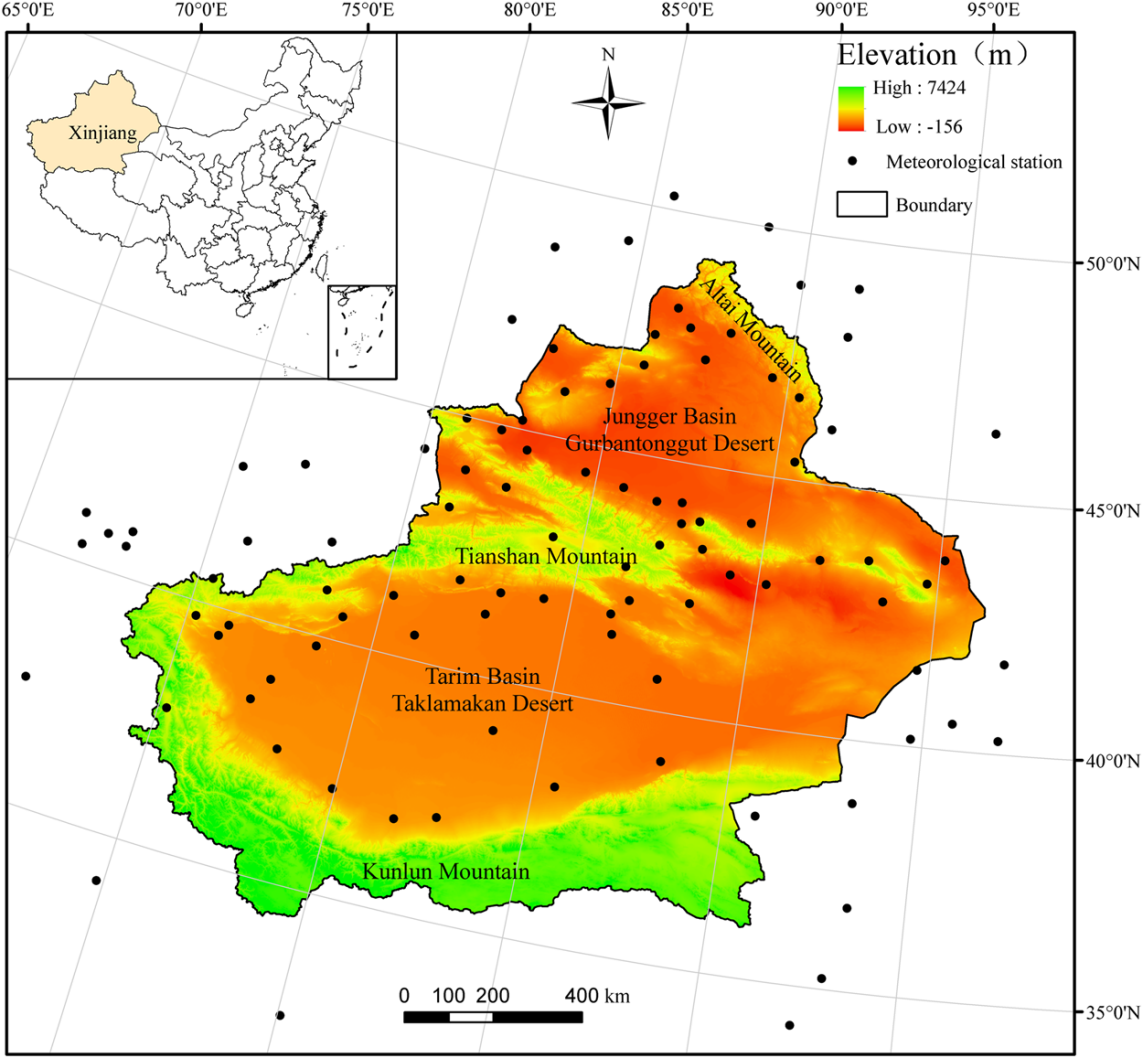
Location of the study area in China and elevation. Black dots represent meterological stations. The maps were generated by ArcGIS 10.2, URL:286 http://support.esri.com/Products/Desktop/arcgis-desktop/arcmap/10-2-2#overview.

Xinjiang Uyghur autonomous region (34°22′–49°33′ N, 73°22′–96°21′ E) is located in northwestern China, with an area of 166 × 10^4^ km^2^, accounting for approximately 1/6 of China. Situated at the center of the Eurasian continent, it is surrounded by high mountains: the Altai Mountains to the north, the Kunlun Mountains to the south, and the Tianshan Mountains in the central part. The Tianshan Mountain cut across northern Xinjiang between the vast Junggar and Tarim basins. These three mountains and two basins form the unique geographical environment (Fig. 4). Xinjiang features a mean annual temperature of 9–12 °C, annual precipitation of 100–200 mm in the north and 16–85 mm in the south, and annual potential evaporation of 1500–2300 mm in the north and 2100–3400 mm in the south. The grassland ecosystems in Xinjiang are mainly distributed across Tianshan Mountain, Altai Mountain, Kunlun Mountain, and along the rivers of Junggar Basin and Tarim Basin.

### Data source

The moderate-resolution imaging spectroradiometer (MODIS) NDVI product (MYD13Q1) with a spatial resolution of 500 m and a temporal scale of 16 days is downloaded from the NASA/EOS LPDAAC data gateway (http://lpdaac.usgs.gov/) from 2000 to 2014. Using the MODIS re-projection tool for mosaic and re-projection processing, the 16-days of NDVI data are composited as a monthly data set using the maximum value composite method for reducing the residual noise caused by haze and clouds. The composited NDVI images are then processed by employing an adaptive Savitzky–Golay smoothing filter to obtain the final monthly NDVI data sets for use.

The meteorological data are obtained from the China meteorological science data-sharing service system (http://data.cma.cn/) and National Environmental Information Center Web site, NOAA, USA (http://www.ncdc.noaa.gov/ghcnm/v3.php). The data includes the monthly average temperature, total precipitation and daily sunshine time recorded by 67 meteorological stations in Xinjiang and 33 meteorological stations in nearby areas from 2000–2014. Thin-plate smoothing spline (ANUSPLIN) interpolation is performed to interpolate the meteorological data for producing raster images with 500 m spatial resolution^36^.

**Figure 5:**
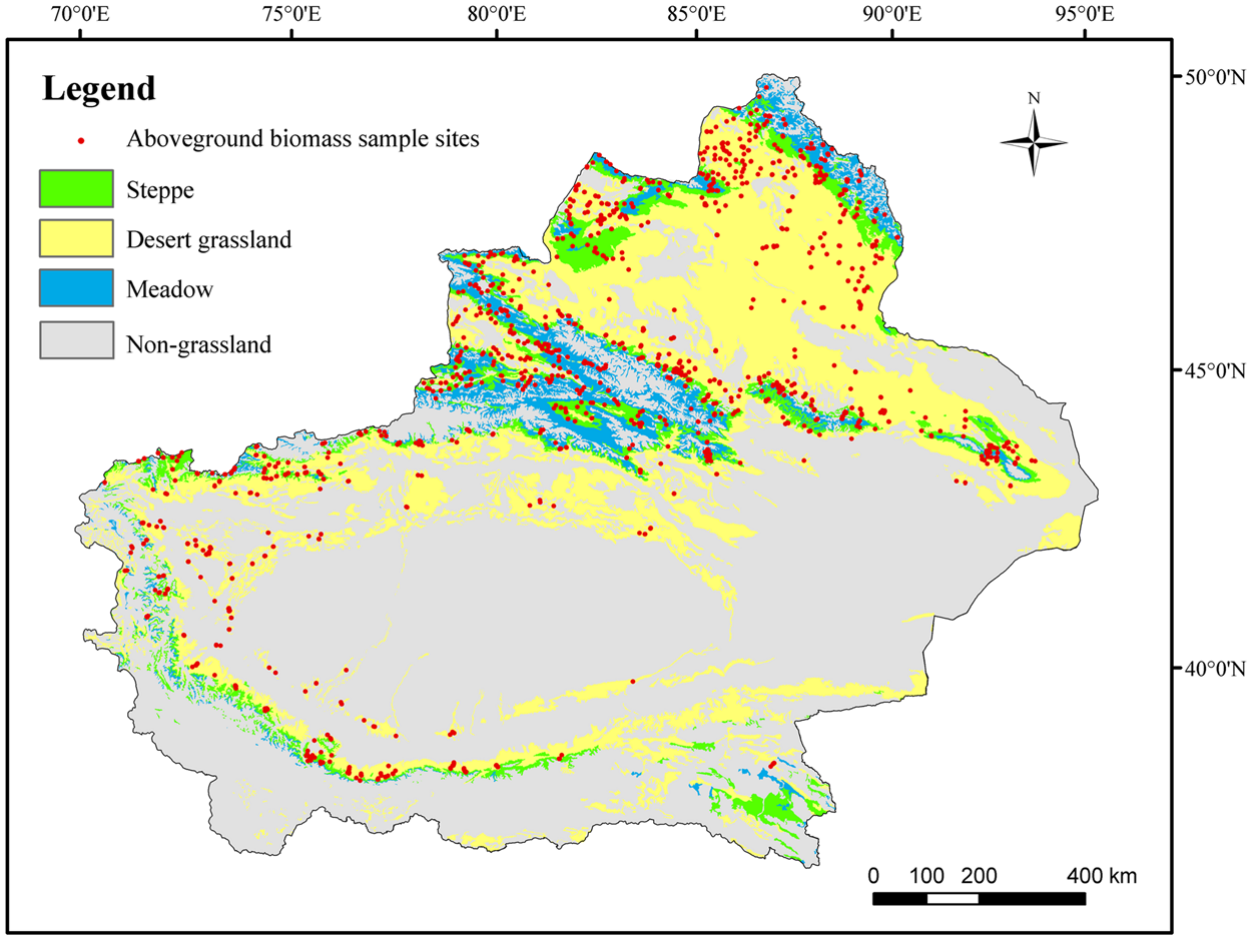
Land cover types and spatial distribution of 791 biomass sample sites (2010–2014) in study area. Red dots represent aboveground biomass sample sites. The maps were generated by ArcGIS 10.2, URL:286 http://support.esri.com/Products/Desktop/arcgis-desktop/arcmap/10-2-2#overview.

The 1:4,000,000 scale grassland type map is obtained from the Natural Resources Comprehensive Investigation Committee of the Chinese Academy of Sciences. The total of 11 grassland types in Xinjiang are grouped into 3 classes according to their properties: Desert grassland (Temperate desert, Temperate steppe-desert and Alpine desert), Steppe grassland (Temperate meadow steppe, Temperate steppe, Temperate desert steppe, Alpine steppe and Alpine desert Steppe) and Meadow grassland (Mountain meadow, Alpine meadow and Lowland meadow) (Fig. 5).

The aboveground biomass data (AGB) (2010–2014) were collected at 791 sampling sites by the General Grassland Station of Xinjiang (Fig. 2) and are downloaded from Ministry of Agriculture Grassland Supervision Center (http://202.127.42.194/jiance/login.aspx). The data were usually collected in late July or August at each grassland site (500 × 500 m). The biomass samples of five quadrates (1 × 1 m) of each site were harvested, and were oven-dried at 65 °C for 48 h to a constant mass and then weighed and averaged as the weight for the site (i.e., AGB); Using the ratio index (i.e., underground biomass divided by aboveground biomass) of each grass type to convert the AGB to total biomass in counting both aboveground and underground biomasses^37^. This total biomass is then converted to NPP (g C/m^2^) by multiply a factor of 0.475^38^ and is then used for validation of the modelled NPP. Table 3 shows a summary of the measured AGB of different types of grassland from the total 791 sampling sites for the period of 2010–2014.

**Table 3:** Measured aboveground biomass of grasslands in Xinjiang, 2010–2014.

Type	AGB (g/m^2^)		Elevation (m)	sample
	Mean	SD		
Steppe	101.1	22.8	1553	372
Meadow	137.7	19.9	1974	245
Desert grassland	54.3	16.5	901	174
All grassland	101.9	20.5	1514	791

### Methods

In this study, we define three types of NPP. The first one is the actual NPP_A_, as calculated by the Carnegie Ames Stanford Approach (CASA) model^39^. The second is climate driven potential NPP_P_ that is calculated by the Miami model^40^. The third is human activity driven NPP_H_^41^, which is the difference between NPP_P_ and NPP_A_:

1 $$NP{P}_{H}=NP{P}_{P}-NP{P}_{A}$$

The Miami Model is a mathematical model that simulates the potential NPP using environmental variables (i.e., annual mean temperature (T, °C) and precipitation (R, mm). This model has been widely used to calculate the climate-driven potential NPP for large areas and at the global scale^28,40^ as shown in equations (2, 3 and 4).

2 $$NP{P}_{P}=\,{\rm{\min }}\,[NP{P}_{T},NP{P}_{R}]$$

3 $$NP{P}_{T}=3000/(1+{e}^{(1.315-0.119T)})$$

4 $$NP{P}_{R}=3000(1-{{\rm{e}}}^{-0.000664R})$$

where NPP_P_ is the potential NPP (g C m^−2^ yr^−1^); NPP_T_ and NPP_R_ are the temperature and precipitation driven potential NPP, respectively; T is the annual mean temperature (°C); and R is the annual precipitation (mm).

The CASA model was used to simulate actual NPP, which is the actual existing NPP that was influenced by both climate change and human activities. The remote sensing and climatic data-based CASA model is a light use efficiency model developed by Potter *et al*.^41^ and it was developed to estimate NPP on a large geographic scale^39,41^. The CASA model is determined by both Absorbed Photosynthetically Active Radiation APAR (MJ/m^2^) and light use efficiency ε (g C/MJ), and is described as follows:

5 $$NPP(x,t)=APAR(x,t)\times \varepsilon (x,t)$$

where x is the spatial location, t is time. APAR (x, t) and ε (x, t) are calculated using Eqs (6) and (7), respectively.

6 $${\rm{A}}PAR(x,t)=SOL(x,t)\times FPAR(x,t)\times 0.5$$

where SOL (x, t) is the total solar radiation (MJ m^−2^) of pixel x in time t, and FPAR (x, t) is the fraction of the photosynthetically active radiation absorbed by vegetation. FPAR (x, t) can be determined by NDVI data^42^; 0.5 represents the proportion of the total solar radiation available for vegetation.

The actual light use efficiency is the efficiency of vegetation absorbed energy into the carbon (C) over dry organic substance, through fixing solar radiation and photosynthesis^41^ and is mainly influenced by temperature and moisture^39^.

7 $$\varepsilon (x,t)={{\rm{T}}}_{\varepsilon 1}(x,t)\times {{\rm{T}}}_{\varepsilon 2}(x,t)\times {{\rm{W}}}_{\varepsilon }(x,t)\times {\varepsilon }_{{\rm{\max }}}$$

where T_ε1_(x, t) and T_ε2_(x, t) denote the temperature stress coefficients on light use efficiency, W_ε_(x, t) is the water stress coefficient that indicates the reduction in light use efficiency caused by moisture, and ε_max_ denotes the maximum light use efficiency under ideal conditions set as the maximum possible light energy conversion efficiency. As for the vegetation in China, it may not be the same as global vegetation parameters^43^; hence, we use the parameters for grassland maximum light use efficiency in China as simulated by Zhu *et al*.^44^ and this term is set uniformly at 0.542 g C MJ^−1^ for grassland in Xinjiang. A more detailed description of algorithm for W_ε_(x, t) calculation and improvement can be found in Zhu *et al*.^44^.

**Figure 6:**
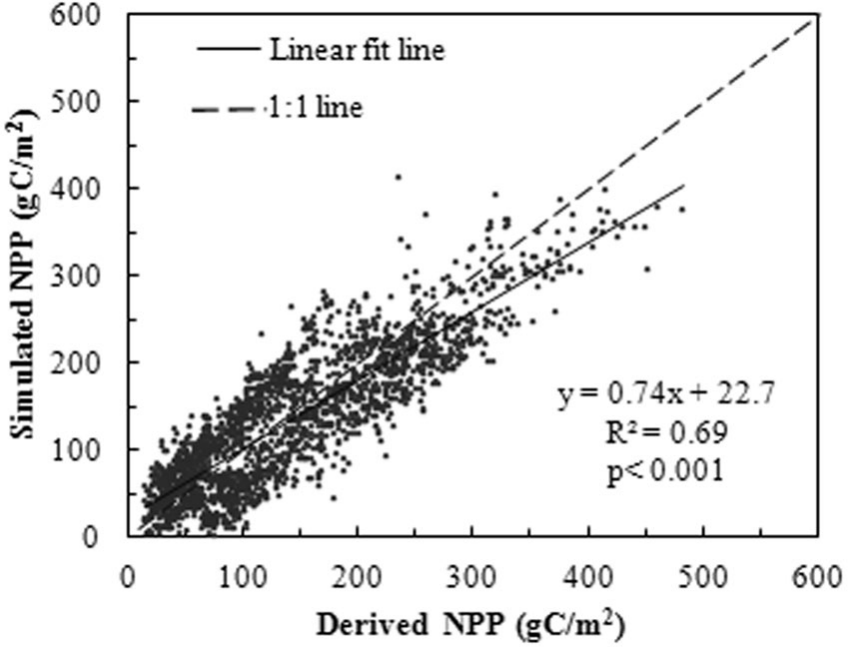
Scatter plot of observed NPP and CASA simulated NPP of Xinjing from 2010 to 2014.

Figure 6 shows the derived NPP (2010–2014), CASA model simulated NPP_A_, and the determination coefficient (R^2^) of 0.69 (P < 0.001), indicating that the CASA model is suitable for estimating the local area’s grassland NPP_A_.

We detect long term annual NPP trend changes in every pixel by linear regression analysis. Existing research shows that the Greenness Rate of Change (GRC) can reflect the change trend of grassland NPP within a certain period of time^45^, following the equation (8):

8 $$SlopeNPP=\frac{n\times {\sum }_{j=1}^{n}(j\times NP{P}_{j})-{\sum }_{j=1}^{n}\,j\,{\sum }_{j=1}^{n}{NPP}_{j}}{n\times {\sum }_{j=1}^{n}\,{j}^{2}-{({\sum }_{j=1}^{n}j)}^{2}}$$

where n is the sequential year; j is numerical order of the year from 2000–2014, and NPP_j_ is the annual NPP in year j. A positive slope value suggests a linear increasing trend (i.e., grassland NPP increase) and vice versa (i.e., grassland NPP decrease). Table 3 shows the relative effects of climate change (SlopeNPP_P_) and human activities (SlopeNPP_H_) on grassland change (SlopeNPP_A_) in six different scenarios.

The nonparametric Mann-Kendall (MK) test is used to estimate the abrupt points for all pixel values for the mean NPP_A_ and NPP_P_ , aggregated over time for the various trend regions. The MK test is a useful exploratory method for identifying monotonic changes during specific time intervals, and has been widely used to test for trends in remote sensing data^46^.
