# Publisher Correction: Grassland dynamics in response to climate change and human activities in Xinjiang from 2000 to 2014

**DOI:** 10.1038/s41598-019-41390-z

**Published:** 2019-04-11

**Authors:** Renping Zhang, Tiangang Liang, Jing Guo, Hongjie Xie, Qisheng Feng, Yusupujiang Aimaiti

**Affiliations:** 1 Institute of Arid Ecology and Environment, Key Laboratory of Oasis Ecology, Xinjiang University, Urumqi, 830046 China; 2 State Key Laboratory of Grassland Agro-ecosystems, College of Pastoral Agriculture Science and Technology, Lanzhou University, Lanzhou, 730020 China; 3 Xinjiang Academy Forestry, Urumqi, 830000 China; 4 Department of Geological Sciences, University of Texas at San Antonio, Texas, 78249 USA; 5 Department of Urban Environment Systems, Chiba University, Chiba, 263-8522 Japan

Correction to: *Scientific Reports* 10.1038/s41598-018-21089-3, published online 13 February 2018

This Article contains an error in the order of the Figures. Figures 1 and 2 were published as Figures 2 and 1 respectively. The correct Figures 1 and 2 appear below. The Figure legends are correct.

**Figure 1:**
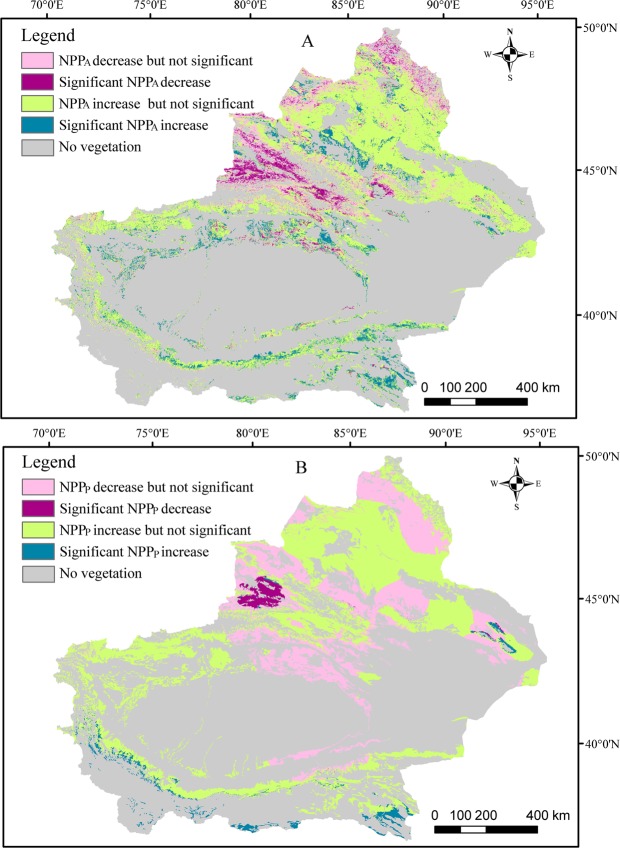
Spatial change trends of Xinjiang grassland NPPA (**a**) and (NPPP) (**b**) from 2000–2014. The maps were generated by ArcGIS 10.2, URL:286 http://support.esri.com/Products/Desktop/arcgis-desktop/arcmap/10-2-2#overview.

**Figure 2:**
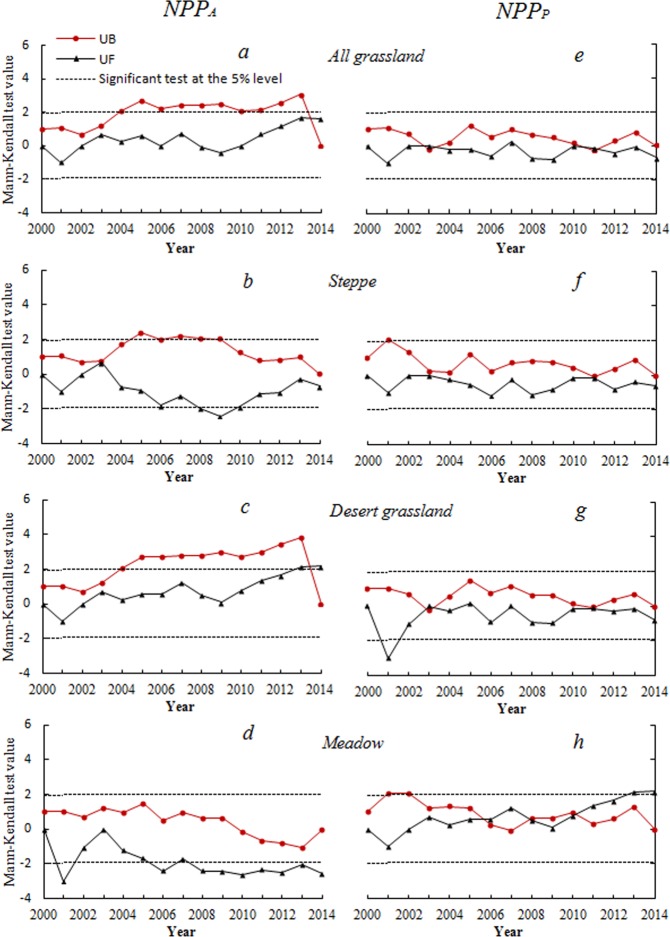
Mann-Kendall mutation criterion curve of average NPPA (left panel) and NPPP (right panel) in Xinjiang grassland from 2000–2014. (**A** and **E**) Mean Mann-Kendall mutation criterion of average NPPA and NPPP of overall grassland; (**B** and **F**) mean Mann-Kendall mutation criterion of average NPPA and NPPP of steppe grassland; (**C** and **G**) mean Mann-Kendall mutation criterion of average NPPA and NPPP of desert grassland; (**D** and **H**) mean Mann-Kendall mutation criterion of average NPPA and NPPP of meadow grassland.

